# Progesterone receptor loss identifies luminal-type local advanced breast cancer with poor survival in patients who fail to achieve a pathological complete response to neoadjuvant chemotherapy

**DOI:** 10.18632/oncotarget.4225

**Published:** 2015-05-22

**Authors:** Sheng Chen, Liang Huang, Can-Ming Chen, Zhi-Ming Shao

**Affiliations:** ^1^ Department of Breast Surgery, Fudan University Shanghai Cancer Center/Cancer Institute, Shanghai, P. R. China; ^2^ Department of Oncology, Shanghai Medical College, Fudan University, Shanghai, P. R. China; ^3^ Institutes of Biomedical Science, Fudan University, Shanghai, P. R. China

**Keywords:** breast cancer, neoadjuvant chemotherapy, survival, progesterone receptor, estrogen receptor

## Abstract

The aim of this study was to investigate the potential of progesterone receptor (PgR) as a biomarker for differentiating estrogen receptor (ER)-positive patients who fail to achieve a pathological complete response to neoadjuvant chemotherapy (NCT) with different prognoses. A total of 327 consecutive, locally advanced breast cancer patients with ER-positive disease were included in this study. According to their HER-2 and Ki-67 status, the patients were classified into the Luminal-A or Luminal-B subtype. We evaluated the clinical and pathological response to NCT and relapse or death occurring during follow-up according to PgR status in the different luminal subtypes. In the Luminal-B subtype, patients with PgR- tumors had a relatively higher pathological complete response (pCR) rate (29.5% *vs*. 4.7% pCR, *P* < 0.001) and Miller-Payne grades (45.5% *vs*. 23.5% of grade 4-5, *P* = 0033) compared to PgR+ tumors. In Luminal-B patients with residual tumor after NCT, PgR loss was also independently correlated with poor relapse-free survival (*P* = 0.017; HR = 0.430; PgR- as a reference) and overall survival (*P* = 0.013; HR = 0.355; PgR- as a reference). However, in the Luminal-A subtype, there were no statistically significant differences between PgR+ and PgR- disease in response to NCT or survival. Our findings have demonstrated the prognostic value of PgR loss in the neoadjuvant setting, indicating that ER+/PgR- Luminal-B tumors warrant further attention due to their high risk of relapse after primary treatment.

## INTRODUCTION

Neoadjuvant chemotherapy (NCT) is the standard of care for local control of local advanced breast cancer (LABC), and allowing breast conservation. Most studies confirmed that patients who achieved a pathological complete response (pCR) after NCT were expected to have a significantly more favorable outcome compared with patients with residual disease in the breast and/or axillary lymph nodes (known as non-pCR) [1, 2]. However, because only 10%-30% of patients experience pCR after primary treatment, the majority of patients still have a high risk of relapse and death. In the last decades, various approaches have focused on differentiating non-pCR responders with different outcomes. Several prognostic models have been reported, and some variables including node status, residual tumor size, Ki-67, hormonal receptor and human epidermal growth factor receptor-2 (HER2) have been shown to be potentially prognostic [3]. However, the results of these studies are not concordant, indicating that the prognostic value of some markers might not be consistent between different study populations.

In recent years, it has become widely accepted that breast cancer can be classified into multiple subtypes by immunohistochemical (IHC) analysis of the estrogen receptor (ER), progesterone receptor (PgR), HER-2, and Ki-67. Such an analysis is considered to be a surrogate means for identifying molecular subtypes of breast cancer with different prognoses across treatment settings [4]. Many common subtypes have been identified, including two that are derived from hormonal receptor (HR)-positive tumors (Luminal-A and Luminal-B) and two that are derived from HR-negative tumors (triple-negative and HER-2+ cancers). The Luminal-type breast cancers, which are often associated with chemoresistance, have a better outcome compared with non-luminal-type breast cancers, as demonstrated by previous data [5, 6]. Moreover, recent studies have suggested that in the neoadjuvant setting, failure to achieve a pCR is clearly associated with worse long-term outcomes in TNBC and HER-2+ breast cancer but not in the majority of hormone receptor–positive breast cancers [7, 8]. Therefore, the difference in the biological features of HR+ and HR- tumors has resulted in different recommendations for adjuvant systemic treatments and is often attributed to the heterogeneity of biomarker studies.

Compared with ER, PgR is often considered to be a weak prognostic marker for determining breast cancer subtype [9]. The absence of PgR may be associated with higher chemosensitivity and anti-estrogen resistance. Recent studies have indicated that ER+, PgR- tumors had more aggressive features that resulted in worse outcomes compared with ER+, PgR+ tumors [10]. However, the prognostic value of PgR in the neoadjuvant setting remains controversial. In the present study, we investigated the relationship between PgR expression and long-term survival in Luminal-type breast cancer patients who received NCT, aiming to identify a distinct subset of ER+ tumors.

## RESULTS

### Patient characteristics and response to NCT

The median age of the 327 patients was 49 years (range: 25-70 years). All patients were diagnosed with stage II or III disease, and 41.0% of them were post-menopausal at diagnosis. A total of 193 patients received an anthracycline-based regimen, 114 received a taxane-based regimen, and 20 received both anthracycline and taxane as an NCT regimen. All the patients had confirmed ER+ disease before NCT, and 225 of them also had positive PgR expression in their primary tumors. According to the expression of HER-2 and Ki-67 in CNB tumor samples as determined by IHC analysis, 198 patients were classified into the Luminal-A subtype, whereas 129 patients were classified into the Luminal-B subtype (56 with HER-2+, 73 with HER-2-). The patient characteristics and responses to NCT by breast cancer subtype are shown in Table 1. Remarkably, patients with Luminal-B disease were more likely to achieve a pCR after NCT, with an observed pCR rate of 13.2%; on the other hand, patients with Luminal-A disease had an observed pCR rate of 5.1% (*P* = 0.009).

**Table 1:** Characteristics of 327 ER+ patients by breast cancer subtype

Characteristics	Total (N=327)		Lumina-A (N=198)		Luminal-B (N=129)		P value
	No.	%	No.	%	No.	%	
Patient age, years							0.737
<50	166	50.8	102	51.5	64	49.6	
≥50	161	49.2	96	48.5	65	50.4	
Menopausal status							0.975
Pre	193	59.0	117	59.1	76	58.9	
Post	134	41.0	81	40.9	53	41.1	
Tumor stage at diagnosis							0.001
T2	116	35.5	76	38.4	40	31.0	
T3	156	47.7	101	51.0	55	42.6	
T4	55	16.8	21	10.6	34	26.4	
LN status at diagnosis							0.609
−	81	24.8	51	25.8	30	23.3	
+	246	75.2	147	74.2	99	76.7	
PgR status							0.358
−	102	31.2	58	29.3	44	34.1	
+	225	68.8	140	70.7	85	65.9	
NCT regimen							0.038
Anthracycline-based	198	60.6	130	65.7	68	52.7	
Taxane-based	103	31.5	52	26.3	51	39.5	
Anthracycline andTaxane	26	8.0	16	8.1	10	7.8	
Clinical response							0.931
CR	35	10.7	21	10.6	14	10.9	
PR	174	53.2	107	54.0	67	51.9	
SD/PD	118	36.1	70	35.4	48	37.2	
Pathological response							0.009
pCR	27	8.3	10	5.1	17	13.2	
Non-pCR	300	91.7	188	94.9	112	86.8	

Abbreviations: LN, lymph node; PgR, progesterone receptor; NCT, neoadjuvant chemotherapy; CR, complete response; PR, partial response; SD/PD, stable disease or progression disease;

Furthermore, in Table 2 we show the distributions of patient responses to NCT among the different subtypes, demonstrating the relationship between PgR and treatment response. In the Luminal-A subtype, there were no statistically significant differences between PgR+ and PgR- disease with respect to clinical response, pathological response according to pCR rate, or response scores according to the MP grading system. However, in the Luminal-B subtype, patients with PgR- tumors had a relatively higher pCR rate (29.5% *vs*. 4.7% pCR, *P* < 0.001) and MP grades (45.5% *vs*. 23.5% grade 4-5, *P* = 0033).

**Table 2:** Patient responses to NCT according to PgR status

Response	Luminal-A (n=198)			Luminal-B (n=129)		
	ALL	PgR-	PgR+	ALL	PgR-	PgR+
***Clinical response***						
**CR**	21（10.6%）	8（13.8%）	13（9.3%）	14（10.9%）	7（15.9%）	7（8.2%）
**PR**	107（54.0%）	33（56.9%）	74（52.9%）	67（51.9%）	23（52.3%）	44（51.8%）
**SD/PD**	70（35.4%）	17（29.3）	53（37.9%）	48（37.2%）	14（31.8%）	34（40.0%）
**P value**		0.415			0.365	
***Pathological response***						
**pCR**	10（5.1%）	6（10.3%）	4（2.9%）	17（13.2%）	13（29.5%）	4（4.7%）
**Non-pCR**	188（94.9%）	52（89.7%）	136（97.1%）	112（86.8%）	31（70.5%）	81（95.3%）
**P value**		0.067*			<0.001	
***MP grading***						
**5-4**	34（17.2%）	13（22.4）	21（15.0%）	40（31.0%）	20（45.5%）	20（23.5%）
**3**	105（53.0%）	31（53.4%）	74（52.9%）	48（37.2%）	14（31.8%）	34（40.0%）
**2-1**	59（29.8%）	14（24.1%）	45（32.1%）	41（31.8%）	10（22.7%）	31（36.5%）
**P value**		0.333			0.033	

Abbreviations: NCT, neoadjuvant chemotherapy; PgR, progesterone receptor; CR, complete response; PR, partial response; SD/PD, stable disease or progression disease; MP, Miller-Payne;

* Fisher's exact test

### Correlation between PgR and survival

Overall, the observed 5-year RFS and 5-year OS of the 327 patients were 68% and 81%, respectively, in the Luminal-A subtype and 67% and 80%, respectively, in the Luminal-B subtype. Failure to achieve a pCR is clearly associated with worse long-term outcomes in Luminal-B breast cancer, although this negative prognostic association is not statistically significant in Luminal-A breast cancers. The 5-year RFS and 5-year OS of patients with Luminal-A patients with pCR were 90.0% (compared with 67.5% in non-pCR patients, log-rank test *P* = 0.168) and 90.0% (compared with 80.4% in non-pCR patients, log-rank test *P* = 0.476), respectively, whereas the 5-year RFS and 5-year OS of Luminal-B patients with pCR were 94.1% (compared with 61.4% in non-pCR patients, log-rank test *P* = 0.018) and 100.0% (compared with 75.9% in non-pCR patients, log-rank test *P* = 0.023), respectively.

**Figure 1:**
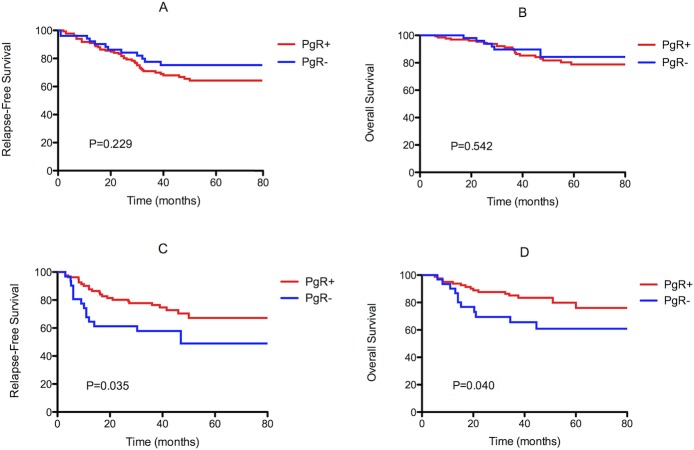
Kaplan-Meier survival curves for relapse-free survival (RFS) and overall survival (OS) of non-pCR patients with Luminal-A (**A, B**) and Luminal-B (**C, D**) primary tumors by PgR status. PgR loss was significantly correlated with poor survival (both RFS and OS) in Luminal-B patients (*P* = 0.035 and *P* = 0.040, respectively), but not in Luminal-A patients (*P* = 0.229 and *P* = 0.542, respectively).

In non-pCR responders, univariate survival analyses were performed separately in the Luminal-A and Luminal-B categories to detect the prognostic value of PgR. PgR loss was significantly correlated with poor survival (both RFS and OS) in Luminal-B patients but not in Luminal-A patients. The distributions of the survival curves are shown in Figure 1A-1D. Additionally, multivariate survival analysis using the Cox regression model was performed in Luminal-B patients. PgR status (*P* = 0.017; HR = 0.430; PgR- as a reference), residual tumor size (*P* = 0.003; HR = 1.690 for 2-5 cm, HR = 3.090 for > 5 cm; < 2 cm as a reference), residual lymph node involvement (*P* = 0.014; HR = 0.843 for 1-3 positive node, HR = 2.624 for ≥4 positive node; 0 positive node as a reference), and Ki-67 (*P* = 0.015; HR = 2.245; < 15% as a reference) were independent prognostic variables for RFS. PgR status (*P* = 0.013; HR = 0.355; PgR- as a reference), residual tumor size (*P* = 0.041; HR = 1.559 for 2-5 cm, HR = 3.664 for > 5 cm; < 2 cm as a reference), and residual lymph node involvement (*P* = 0.014; HR = 0.949 for 1-3 positive node, HR = 3.408 for ≥4 positive node; 0 positive node as a reference) were also independent prognostic variables for OS. The results of the univariate and multivariate survival analyses are shown in Table 3.

**Table 3:** Univariate and multivariate survival analysis for Luminal-B patients with residual disease after NCT

Variable	RFS				OS			
	Unv.	Muv.			Unv.	Muv.		
	P	Harzard ratio	95% CI	P	P	Hazard ratio	95%CI	P
Patient age, years	0.153	-	-	-	0.371	-	-	-
<50								
≥50								
Menopausal status	0.419	-	-	-	0.707	-	-	-
Pre								
Post								
NCT regimen	0.281	-	-	-	0.532	-	-	-
Anthracycline-based								
Taxane-based								
Anthracycline and Taxane								
Primary tumor stage	0.108	-	-	-	0.107	-	-	-
T2								
T3								
T4								
Primary LN status	0.123	-	-	-	0.067	-	-	-
-								
+								
PgR status	0.035			0.017	0.040			0.013
-		Ref.					Ref.	
+		0.430	0.215-0.858				0.355	0.157-0.801
HER-2 status	0.275	-	-	-	0.141	-	-	-
-								
+								
Residual tumor (cm)	0.003			0.046	0.004			0.041
<2		Ref.					Ref.	
2-5		1.690	0.727-3.931				1.559	0.540-4.499
>5		3.090	1.274-7.491				3.664	1.276-10.519
Residual LN involvement	0.010			0.013	0.014			0.027
0		Ref.					Ref.	
1-3		0.843	0.233-3.049				0.949	0.152-5.929
4+		2.624	0.893-7.712				3.408	0.769-15.096
Residual tumor grade	0.027	-	-	0.659	0.175	-	-	-
I-II								
III								
Vascular invasion	0.623	-	-	-	0.851	-	-	-
Yes								
No								
Residual tumor Ki-67	0.011			0.015	0.403	-	-	-
<15%		Ref.						
≥15%		2.245	1.172-4.298					
CT	0.619	-	-	-	0.258	-	-	-
Anthracycline								
Taxane								
Anthracycline and Taxane								
None								
RT	0.372	-	-	-	0.200	-	-	-
Yes								
No								
ET	0.611	-	-	-	0.359	-	-	-
Yes								
No								

Abbreviations: Unv, univariate analysis; Muv, multivariate analysis; Ref, reference; LN, lymph node; NCT, neoadjuvant chemotherapy; PgR, progesterone receptor; HER-2, human epidermal receptor-2; CT, chemotherapy; RT, radiotherapy; ET, endocrinotherapy

**Figure 2:**
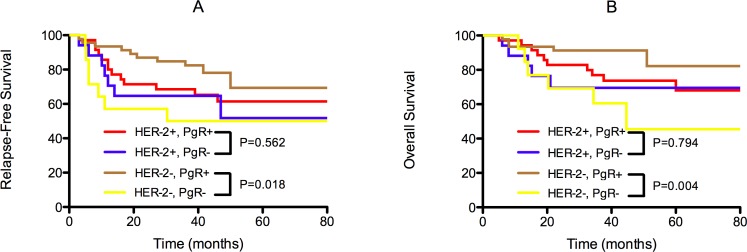
Kaplan-Meier survival curves for relapse-free survival (RFS) and overall survival (OS) by PgR status and HER-2 status in non-pCR patients with Luminal-B primary tumors. PgR was significantly correlated with RFS and OS in HER-2- Luminal-B patients (*P* = 0.018 and *P* = 0.004, respectively) but not in HER-2+ Luminal-B patients (*P* = 0.562 and *P* = 0.794, respectively).

In addition, PgR had different prognostic values for patient survival with respect to HER-2 status (Figure 2A, 2B). It was significantly correlated with RFS and OS in HER-2- Luminal-B patients (*P* = 0.018 and *P* = 0.004, respectively). However, in HER-2+ Luminal-B patients, there was no significant difference in either RFS or OS with respect to the PgR category (*P* = 0.562 and *P* = 0.794, respectively).

## DISCUSSION

NCT, which downstages the disease and enables surgery to be performed in those initially deemed inoperable, has been widely accepted as a standard of care for LABCs. Most published studies have affirmed that patients who achieve pCR, irrespective of the initial stage and molecular subtype, have a very low risk of relapse and death [2, 11]. However, a pCR occurs in only approximately 10%-30% of all cases and depends on the biological features of the primary tumor to a large extent. For patients who fail to achieve pCR, the response to subsequent treatment and prognosis also varies between different subgroups [12, 13].

Although gene expression profiling has led to a better understanding of the biological phenotypes of breast cancer, its technical complexity and high costs have limited its clinical application. The combination of IHC markers, including ER, PgR, HER-2, and Ki-67, has been accepted as a substitute for molecular subtypes, although the results obtained with gene expression profiling and IHC do not exactly correspond [14, 15]. It is well known that HER-2 overexpression, negativity for hormonal receptors, and a high Ki-67 index are significantly correlated with high chemosensitivity and result in different responses to chemotherapy [16]. Therefore, breast cancer subtypes as defined by differential ER, PgR, HER-2, and Ki-67 staining may display different response rates and different prognoses in the neoadjuvant setting. For ER+ breast cancers, neoadjuvant endocrine therapy other than chemotherapy might be a more appropriate choice for Luminal-A LABCs; however, there is a lack of consensus on the threshold indication for inclusion of NCT for the Luminal-B subtype. Compared to Luminal-A breast cancer, the Luminal-B phenotype has aggressive clinical and biological features and is characterized by lower levels of ER-related genes, higher levels of HER-2-associated genes, and higher levels of proliferative markers [17, 18].

Given that HER-2 overexpression was only observed in 30% of Luminal-B tumors, the Luminal-B phenotype still represents a heterogeneous group of breast cancers. A better understanding of this tumor phenotype might allow better determination of appropriate diagnostic and therapeutic strategies. Several approaches have been undertaken to differentiate Luminal-B tumors with different chemosensitivity and survival [19, 20]. Recently, G. Cancello [21] reported a relatively good prognosis in the PgR+ subgroup of Luminal-B patients and highlighted the significant impact of PgR status on the outcome of patients with early breast cancer. The author claimed to consider more chemotherapy in the ER+/PgR−/HER-2− subgroup and less radical therapy for the “triple positive” subgroup in the primary setting. It is speculated that ER+/PgR- tumors may display more aggressive features than ER+/PgR+ tumors. In this study, we also found that PgR was an important predictive and prognostic biomarker in Luminal-B patients in the neoadjuvant setting. Compared with PgR+ patients, patients with PgR- Luminal-B disease had relatively higher pCR rates and MP grades. However, among Luminal-B patients failing to achieve pCR, the loss of PgR resulted in a high risk of relapse or death. This paradox indicated that PgR status could be used to identify a unique phenotype of Luminal-B breast cancers. This group of tumors has higher response rates to neoadjuvant chemotherapy; however, this advantage is not clearly translated into survival benefit among non-pCR patients.

In this study, the discrimination of Luminal-A and Luminal-B subtype were based on the criteria of St.Gallen Consensus 2011. However, in 2013, some experts suggested that ER+/PgR-low patients should also be included in the Luminal-B subtype based on gene expression profiling. Interestingly, in this study, PgR was only prognostic in HER-2-/Luminal-B tumors but not in HER-2+/Luminal-B or Luminal-A ER+ tumors, which indicated that PgR-/HER-2- with low Ki-67 could not be simply affiliated to Luminal-B subtype.

Considering their high response rate to chemotherapy and poor survival, ER+/PR-/HER-2- patients with high proliferation rates displayed biological behaviors similar to those of patients with triple-negative breast cancer [12]. Despite a better response to neoadjuvant treatments compared to other subtypes of tumors, the long-term prognosis of PR-/HER-2- Luminal-B patients is worse overall than that of other subsets, particularly in the first three years after treatment (Figure 2). Thus, Luminal-B tumors that are resistant to primary chemotherapy might exhibit aggressive behavior and benefit little from further chemo-endocrine treatment.

The loss of PgR may be a surrogate marker of a nonfunctional ER, and it also reflects aberrant growth factor signaling that activates other ER functions other than the PgR pathway [22]. Thus, lack of PgR may contribute to endocrine resistance, which results in poor outcome. Furthermore, ER+/PgR- tumors might also express higher levels of HER-1 and HER-2, which are associated with a significantly poorer outcome, whereas in ER+/PgR+ disease, neither HER-1 expression nor HER-2 overexpression is associated with outcome [22]. In this study, PgR failed to show prognostic value in HER-2+ patients. Because trastuzumab was not given prior to surgery in this study, HER-2+ patients who did not achieve pCR could still benefit from adjuvant trastuzumab, which might result in an unclear survival impact of PgR loss in this specific phenotype. Thus, additional studies examining ER+/HER-2+ patients treated with trastuzumab prior to surgery are needed to clarify this issue.

In summary, although Luminal-type patients are assumed to have a relatively good prognosis, different permutations and combinations of biomarkers are associated with variable tumor behavior, as we have described for the ER+/PR−/HER-2- subset. Our findings have demonstrated the prognostic value of PgR loss in the neoadjuvant setting, indicating that PgR- high Luminal-B tumors require greater attention due to their high risk of relapse after primary treatment. Prospective approaches regarding tailored treatment strategies should be considered for this unique subset of Luminal-B tumors.

## PATIENTS AND METHODS

### Study population

The patient cohort in the present study had ER+ primary breast cancer and received NCT followed by surgery at Shanghai Cancer Center from 1999 to 2009. All patients’ diseases were confirmed as invasive carcinoma through core needle biopsy (CNB), and node status was assessed through fine needle aspiration (FNA) of palpable lymph nodes before NCT. Patients who had any treatment prior to NCT were not eligible for this study. Other exclusion criteria included metastatic disease before surgery, bilateral breast cancer, male breast cancer, and inflammatory breast cancer. Informed written consent was obtained from all subjects prior to the study.

A total of 327 consecutive locally advanced breast cancer patients met the above criteria. The NCT regimens included anthracycline-based, taxane-based, and anthracycline-taxane-based regimens for a median of 3 cycles, as previously reported [23]. For all patients, the surgical procedure included mastectomy and axillary lymph node dissection upon the completion of NCT. Additional cycles of chemotherapy were subsequently performed to complete a total of 6–8 cycles followed by radiation therapy at the discretion of the treating physician and radiologist. Endocrine therapy was recommended for all patients in the present study. Due to health insurance-related limitations, trastuzumab was not utilized before surgery in patients overexpressing HER-2.

Responses to NCT were evaluated clinically and pathologically. Clinical response was based on the reduction of tumor size and node status as detected through MRI and ultrasound according to Response Evaluation Criteria in Solid Tumors (RECIST) 1.1 [24]. Pathological response was evaluated according to the Miller-Payne (MP) grading system [25]. A pathological complete response (pCR) after NCT was defined as the absence of invasive carcinoma in both the breast tissue (MP Grade 5) and lymph nodes of the resected specimen. Patients with ductal carcinoma in situ (DCIS) only were also considered as pCR responders.

### Immunohistochemistry and intrinsic subtypes

Immunohistochemistry and/or fluorescence in situ hybridization (FISH) assays were used for the detection of ER, PgR, HER-2 and Ki-67. The cut-off value for ER and PgR positivity was 1% tumor cells with positive nuclear staining. HER-2 was evaluated as 0, 1+, 2+ or 3+ using circumferential membrane-bound staining, and positivity (HER-2+) was considered as 3+ based a positive result via IHC analysis or positivity via FISH; cases considered as 0 to 1+ or 2+ without FISH positivity were regarded as negative (HER-2-). The Ki-67 value was expressed as the percentage of positive cells (at least 1000) with nuclear staining in each case. The antibodies used were as follows: ER (M7047, clone 1D5, Dako, Denmark), PgR (M3569, clone PgR 636, Dako), HER-2 (A0485, polyclonal rabbit antibody, Dako), and Ki-67 (M7240, clone MIB-1, Dako). On the basis of the 2011 St. Gallen consensus [15], the patient cohort of the present study was divided into two intrinsic subtypes: Luminal-A (HER-2 negative and Ki-67 < 15%) and Luminal-B (HER-2 positive, or HER-2 negative and Ki-67≥15%).

### Statistical analysis

The chi-square test was used to evaluate the distribution of patient characteristics and the relationship between PgR and response to NCT among Luminal-A and Luminal-B subtypes. The Fisher exact test was performed when necessary. Survival analyses were performed using the Cox regression model. Statistically significant prognostic variables in the univariate analysis were tested in the multivariate model with forward selection. The distributions of survival curves were shown using the Kaplan–Meier method, and the differences were measured using the log-rank test. Relapse-free survival (RFS) was calculated from the date of surgery to the date of disease relapse (local or distant relapse or death from any cause). Overall survival (OS) was calculated from the date of diagnosis to the date of death or last follow-up. Patients without events or death were censored at the last follow-up. All P-values reported were two sided and were calculated at a significance level of 0.05. All statistical procedures were carried out using SPSS (version 13.0; SPSS Company, Chicago, IL).
